# *Rnf32* is not essential for spermatogenesis and male fertility in mice

**DOI:** 10.7717/peerj.19794

**Published:** 2025-07-30

**Authors:** Hao Kong, Yufeng Yin, Ni Zeng, Yunfei Zhu, Yiqiang Cui

**Affiliations:** State Key Laboratory of Reproductive Medicine and Offspring Health, Nanjing Medical University, Nanjing, China

**Keywords:** Rnf32, Spermatogenesis, Fertility, Testis, CRISPR/Cas9, Mice

## Abstract

**Background:**

Ring finger motifs are found in a variety of proteins with diverse functions, often involved in protein-DNA or protein–protein interactions. The *Rnf32*-encoded protein contains two such motifs and is predominantly expressed in the testes and ovaries, suggesting that its expression may be regulated by elements within the *Rnf32* promoter region. *Rnf32* is active during spermatogenesis, mainly in spermatocytes and spermatids, indicating a potential role in sperm development.

**Methods:**

We established an *Rnf32* knockout (*Rnf32*^−/−^) mouse model using CRISPR/Cas9 technology. Gene expression was analyzed *via* reverse transcription quantitative polymerase chain reaction (RT-qPCR). Testicular and epididymal phenotypes were assessed through histological and immunofluorescence staining, and fertility and sperm motility were evaluated.

**Results:**

Here, we successfully established an *Rnf32* knockout mouse model using CRISPR/Cas9 technology. Surprisingly, male *Rnf32*^−/−^ mice exhibited normal fertility, with no significant differences in testicular and epididymal histology, spermatogenesis, sperm count, or motility compared to *Rnf32*^+/+^ mice. These findings suggest that *Rnf32* may not be essential for male fertility in mice, and its potential functions warrant further investigation.

## Introduction

Recent epidemiological studies indicate that infertility affects nearly 15% of reproductive-age couples in China, with a persistent upward trend observed over the past two decades (De Kretser & Baker, 1999; Hackstein, Hochstenbach & Pearson, 2000). Male-related factors account for over 50% of these cases, predominantly linked to disrupted spermatogenic processes (Barratt et al., 2017). Clinically, such dysregulation manifests as compromised sperm parameters, including reduced counts, impaired motility, and morphological defects (McLachlan et al., 2007). Therefore, understanding the molecular mechanisms governing germ cell development represents a critical frontier in addressing male reproductive disorders. Spermatogenesis in mammals such as mice is a highly coordinated process of germ cell development, predominantly occurs in the seminiferous tubules through three key phases: spermatogonial stem cell self-renewal and differentiation, meiotic division of spermatocytes, and post-meiotic spermiogenesis involving cytoplasmic remodeling (Chen et al., 2018). Mature sperm released into the tubular lumen undergo functional maturation during epididymal transit, acquiring progressive motility and fertilization competence (Cooper, 2007; Dacheux & Dacheux, 2014). Final activation events, including capacitation and acrosome reaction, occur in the female reproductive tract to enable successful oocyte fusion (Ickowicz, Finkelstein & Breitbart, 2012; Saint-Dizier et al., 2020).

RPL39L, a germline-specific ribosomal protein, exhibits stage-specific expression in pachytene spermatocytes and post-meiotic germ cells (round/elongated spermatids). Phenotypic analysis of RPL39L-deficient mice revealed impaired sperm quality parameters (morphology, count, and motility), confirming its essential role in male reproductive competence. Proteomic analysis further revealed that the silencing of RPL39L induces the coordinated downregulation of 276 cellular proteins (Zou et al., 2021; Li et al., 2022). Among these downregulated proteins, we specifically focused on *Rnf32*, hypothesizing that it may also be a component of the ribosomal or translational regulatory network and is specifically expressed in the testis. Given its potential role and testis-specific expression, we propose that *Rnf32*, like RPL39L, may play a critical role in male reproduction. This chromosome 7q36-located gene encodes a 362-amino acid protein containing two RING-H2 zinc-binding domains flanking a central IQ calmodulin-binding motif. Notably, *Rnf32* represents the first reported gene encoding a dual RING-H2 domain protein in mammals (Van Baren et al., 2002), positioning it as a unique model for investigating ubiquitination-related regulatory networks in reproductive biology.

Transcript analysis of *Rnf32* splice variants revealed distinct functional impacts: Exon 1 variations maintain the open reading frame (ORF) due to the initiation codon’s exclusive localization in exon 2. However, a premature termination codon in exon 3a causes protein truncation upstream of the first RING domain. Furthermore, extended exon 4 transcripts contain a 16-base termination signal immediately downstream of the canonical splice site, resulting in ORF truncation within the first RING-H2 domain and producing a 145-amino acid polypeptide (Van Baren et al., 2002; Chasapis & Spyroulias, 2009). Tissue-specific expression profiling demonstrated *Rnf32*’s predominant transcription in gonadal tissues, with stage-specific upregulation during spermatogenesis. This spatiotemporal expression pattern, particularly in meiotic spermatocytes and post-meiotic germ cells, strongly suggests its functional involvement in gamete maturation (Yu et al., 2003; Guo et al., 2004). Despite our existing understanding of *Rnf32*, its specific role in spermatogenesis remains enigmatic. To further explore this issue, this study adopted the advanced CRISPR/Cas9 technology and successfully constructed a *Rnf32* gene knockout mouse model. With the help of this model, we hope to unambiguously reveal the physiological role played by *Rnf32* in the complex mechanism of spermatogenesis.

## Materials & Methods

### Animal experiments

All experimental animals were obtained from the Animal Core Facility of Nanjing Medical University. The animal experimentation involving C57BL/6 mice adhered to the guidelines established by the Institutional Animal Care and Use Committee (IACUC) of Nanjing Medical University (IACUC-2307030). The experiments were conducted under specific pathogen-free (SPF) conditions, with a light cycle of 12/12 h, a relative humidity ranging from 50 to 55%, and an ambient temperature maintained at (23 ± 2) °C. Throughout the study, the animals had unrestricted access to autoclaved feed and sterile water, and humane endpoints were strictly observed. The mice were randomly assigned to cages, with each cage accommodating 4–5 mice. All cages were maintained under similar conditions, including cage density, bedding, and cleaning frequency. To harvest testicular and epididymal tissues from adult mice for subsequent experimental analyses, our procedures adhered to the 2020 edition of the American Veterinary Medical Association’s Guidelines for the Euthanasia of Animals. Euthanasia was performed using compressed carbon dioxide (CO_2_) delivered into a rodent euthanasia chamber. The CO_2_ flow rate was calibrated to displace 30%–70% of the chamber volume per minute, ensuring a consistent and controlled introduction of the gas. This method rapidly induced unconsciousness in the animals, thereby minimizing potential distress. Following the cessation of breathing, CO_2_ administration was maintained for at least one additional minute to confirm euthanasia. This approach aligns with established protocols in life sciences research, prioritizing both ethical considerations and experimental reproducibility. The *Rnf32* knockout model (C57BL/6N background) was established through CRISPR/Cas9-mediated embryonic genome editing. Two sgRNAs (sgRNA-1: 5′-gatattggttctttctacagagg-3′; sgRNA-2: 5′-aacaagagtgatacatgatgggg-3′) were co-delivered with Cas9 mRNA into zygotes *via* microinjection. We initially introduced Cas9 and sgRNA into embryos to obtain F0 generation mice. Through subsequent breeding for three generations, we successfully established a stable knockout mouse line.

### RNA extraction and quantitative real-time PCR

Total RNA isolation from *Rnf32*^+/+^ (*n* = 3) and *Rnf32*^−/−^ (*n* = 3) murine specimens was performed using TRIzol reagent (15596026; Thermo Fisher Scientific) following standard phenol-chloroform extraction protocols. Complementary DNA (cDNA) synthesis was carried out with the PrimeScript™ Reverse Transcriptase kit (RR037A; Takara Bio) according to the manufacturer’s protocol. The resultant cDNA served as template for both conventional reverse transcription-PCR (RT-PCR) using 2 × RapidTaq Master Mix (P515; Vazyme Biotech) and quantitative real-time PCR (RT-qPCR) with AceQ™ SYBR Green Master Mix (Q131; Vazyme Biotech). Amplification was conducted on a QuantStudio 5 Real-Time PCR system (A28573; Applied Biosystems) under optimized thermal cycling parameters: initial denaturation at 95 °C for 10 min, followed by 40 cycles of denaturation (95 °C, 10 s), annealing (65 °C, 1 min), signal acquisition (97 °C, 1 s), and final extension (37 °C, 30 s). In all reverse transcription quantitative polymerase chain reaction (RT-qPCR) assays, 18S ribosomal RNA (rRNA) was employed as the endogenous control to ensure accurate normalization of gene expression data. Primers were obtained from PrimerBank (ID:31981166a1), which provides comprehensive validation data including QPCR Validation Results, Amplification Plots, and Dissociation Curves. Primer sequences are detailed in Table S1.

### Fertility test

To evaluate reproductive competence, C57BL/6J adult male mice (8–10 weeks old) of *Rnf32*^+/+^ and *Rnf32*^−/−^ genotypes (*n* = 3 per group) were housed in monogamous mating pairs with three sexually mature *Rnf32*^+/+^ females (postnatal day 56). A restricted 12-hour nocturnal mating window was implemented, during which individual male–female pairs were cohabited. Copulatory plug formation was verified post-coitum as a primary fertilization indicator, followed by longitudinal tracking of parturition outcomes to quantify litter size. This experimental paradigm was triplicated using independent male cohorts to ensure statistical robustness.

### Harvesting of tissues and histological analysis

Testicular and epididymal tissues were promptly dissected following euthanasia of the adult male mice (8–10 weeks old). Specimens underwent fixation in modified MDF fixative (24 h minimum), followed by graded ethanol dehydration and paraffin embedding. Serial sections (five µm thickness) were prepared using a rotary microtome and mounted on poly-L-lysine-coated slides. Deparaffinized sections were subjected to hematoxylin and eosin (H&E) staining for morphological assessment using standardized histopathological protocols. All tissues collected from unmated males.

### Sperm motility and sperm count assays

Following euthanasia of the mice, epididymal tissues were microsurgically dissected from adult C57BL/6 mice (postnatal day 56; *n* = 3) and immediately immersed in modified human tubal fluid (HTF) medium (#90126; Irvine Scientific) supplemented with 10% fetal bovine serum. The cauda epididymis was incised under sterile conditions to release spermatozoa into the medium, followed by a 5-min incubation at 37 °C. Computer-assisted sperm analysis (CASA; Hamilton Thorne IVOS II) was subsequently performed to quantify sperm concentration and kinematic parameters. All tissues collected from unmated males.

### Immunofluorescence

Paraffin-embedded testicular sections (five µm thickness) underwent sequential deparaffinization in xylene, rehydration through graded ethanol series, and heat-mediated antigen retrieval in 10 mM sodium citrate buffer (pH 6.0) at 95 °C for 15 min. Non-specific binding was blocked with 5% BSA in PBS (2 h, RT) prior to overnight incubation with primary antibodies at 4 °C. After three PBST (0.05% Tween-20) washes, sections were incubated with Alexa Fluor-conjugated secondary antibodies (2 h, RT) and counterstained with Hoechst 33342 nuclear dye (Invitrogen; 5 min). Fluorescent images were acquired using a Zeiss LSM800 confocal system with ZEN imaging software.

### TUNEL

Apoptotic activity was evaluated using terminal deoxynucleotidyl transferase dUTP nick-end labeling (TUNEL) assay (Cat# A112; Vazyme Biotech) according to standardized protocols. Paraffin-embedded testicular sections (five µm thickness) underwent sequential processing including rehydration, proteinase K digestion (20 µg/ml, 15 min), and enzymatic labeling with TUNEL reaction mixture (37 °C, 1 h). Fluorescence imaging was performed using a Zeiss LSM 880 confocal system (Oberkochen, Germany) equipped with Airyscan detection. Quantitative assessment of germ cell apoptosis was conducted by systematically analyzing fifty seminiferous tubule cross-sections per biological replicate, with apoptotic index calculated as TUNEL-positive cells per tubular circumference.

### Statistical analysis

Quantitative data are presented as mean ± SEM. Statistical analyses of inter-genotypic differences were performed using two-tailed unpaired Student’s *t*-tests implemented in GraphPad Prism v9.0 (GraphPad Software, La Jolla, CA, USA). Significance thresholds were defined as follows: ^∗^*P* < 0.05, ^∗∗^*P* < 0.01, ^∗∗∗^*P* < 0.001, and ^∗∗∗∗^*P* < 0.0001, with “ns” indicating non-significance.

## Results

### The expression of *Rnf32* and the generation of *Rnf32*^−/−^ mice

**Figure 1:**
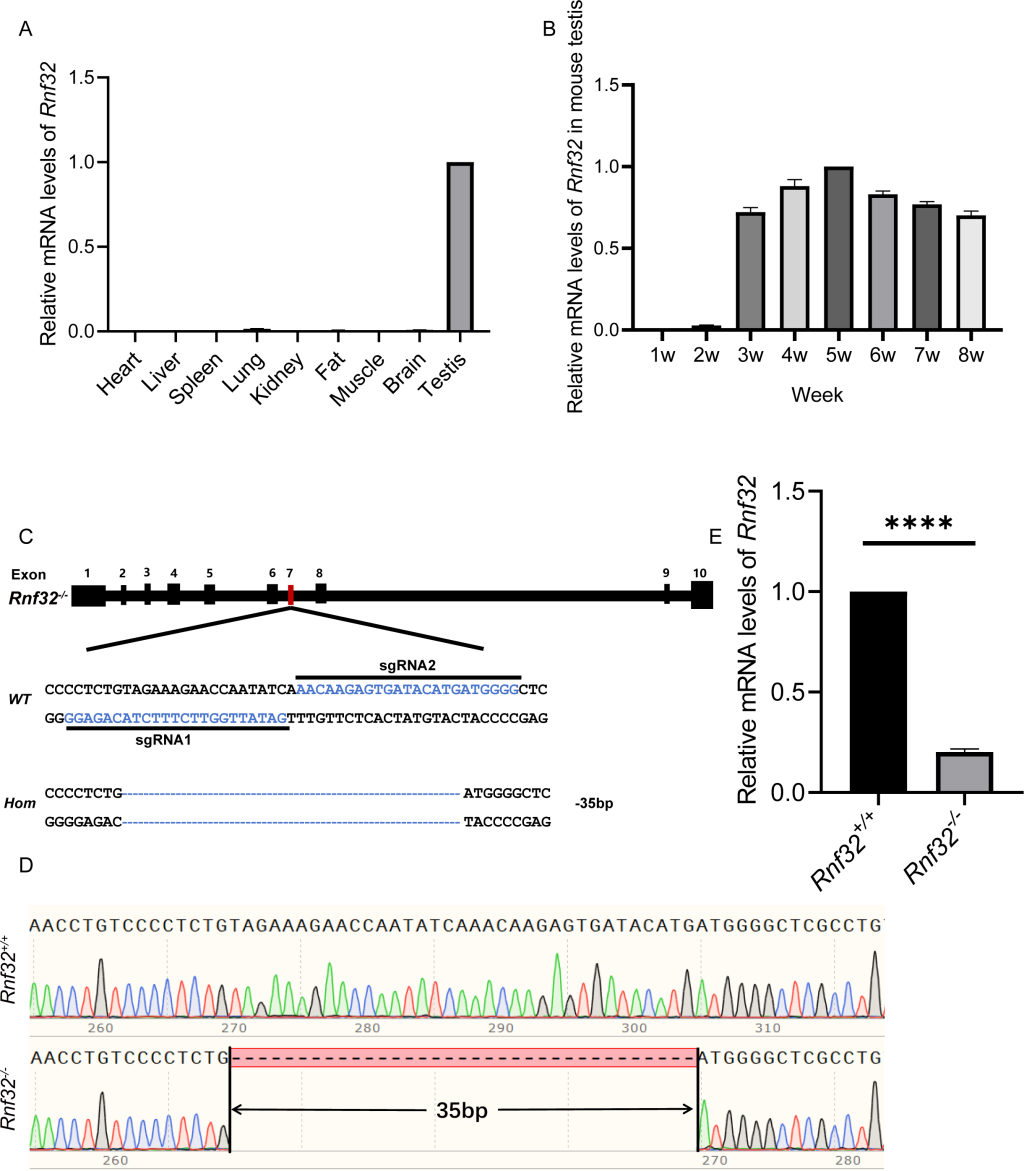
The expression of *Rnf32* and the generation of *Rnf32*^−/−^ mice. (A) Tissue-specific *Rnf32* transcript profiling by RT-PCR analysis. mRNA levels were quantified across multiple murine organs, with data normalized to 18S rRNA endogenous control (*n* = 3). (B) Developmental-stage-specific expression profiles of * Rnf32* in testicular tissue. Temporal dynamics were assessed at defined postnatal intervals (*n* = 3). (C) CRISPR-Cas9-mediated homologous recombination strategy for *Rnf32* knockout. Schematic illustrates sgRNA target sites flanking exon 7. Electropherogram analysis verified a 35-bp frameshift deletion (red box) inducing a premature termination codon. (D) Validation of the *Rnf32*^−/−^ allele by bidirectional Sanger sequencing. Chromatogram depicts precise genomic deletion (dashed line) in mutant mice. (E) Comparative transcript analysis of *Rnf32* mRNA in wild-type *versus Rnf32*^−/−^ testicular tissue, *n* = 3, ^∗∗∗∗^*P* < 0.0001.

*Rnf32* is highly conserved in mammals (Van Baren et al., 2002). Therefore, studying its reproductive effects on mice provides critical insights into human reproductive pathologies. To explore its biological roles, we first performed quantitative PCR (qPCR) to analyze tissue-specific *Rnf32* expression profiles in mice. This analysis identified predominant *Rnf32* expression within the male reproductive system, with testicular tissue showing significantly higher transcript levels compared to other examined organs (Fig. 1A). Developmental time-course analysis revealed stage-specific upregulation, with *Rnf32* expression peaking at postnatal week 3 (Fig. 1B), a critical period corresponding to spermatid differentiation during murine testicular maturation (Gong et al., 2013; Nishimura & L’Hernault, 2017; Chen et al., 2024). To mechanistically investigate *Rnf32*’s role in gametogenesis, we engineered a CRISPR/Cas9-mediated exon 7 in-frame deletion (Fig. 1C) through microinjection of sgRNA/Cas9 complexes into superovulated zygotes. Exon 7 was selected as the target site because it encodes a critical structure motif within the zinc finger domain in the *Rnf32*. By specifically targeting this domain for disruption, we have effectively compromised the structural integrity and consequently maximally impaired the functional capacity of this protein. Sanger sequencing confirmed successful generation of *Rnf32*^−/−^ mice, revealing a precise 35-bp deletion within exon 7 (Fig. 1D). Transcript analysis *via* RT-PCR confirmed efficient *Rnf32* knockout, demonstrating significantly reduced mRNA levels in testicular tissue from *Rnf32*^−/−^ animals compared to wild-type controls (Fig. 1E).

### *Rnf32*^−/−^ mice are fertile

**Figure 2:**
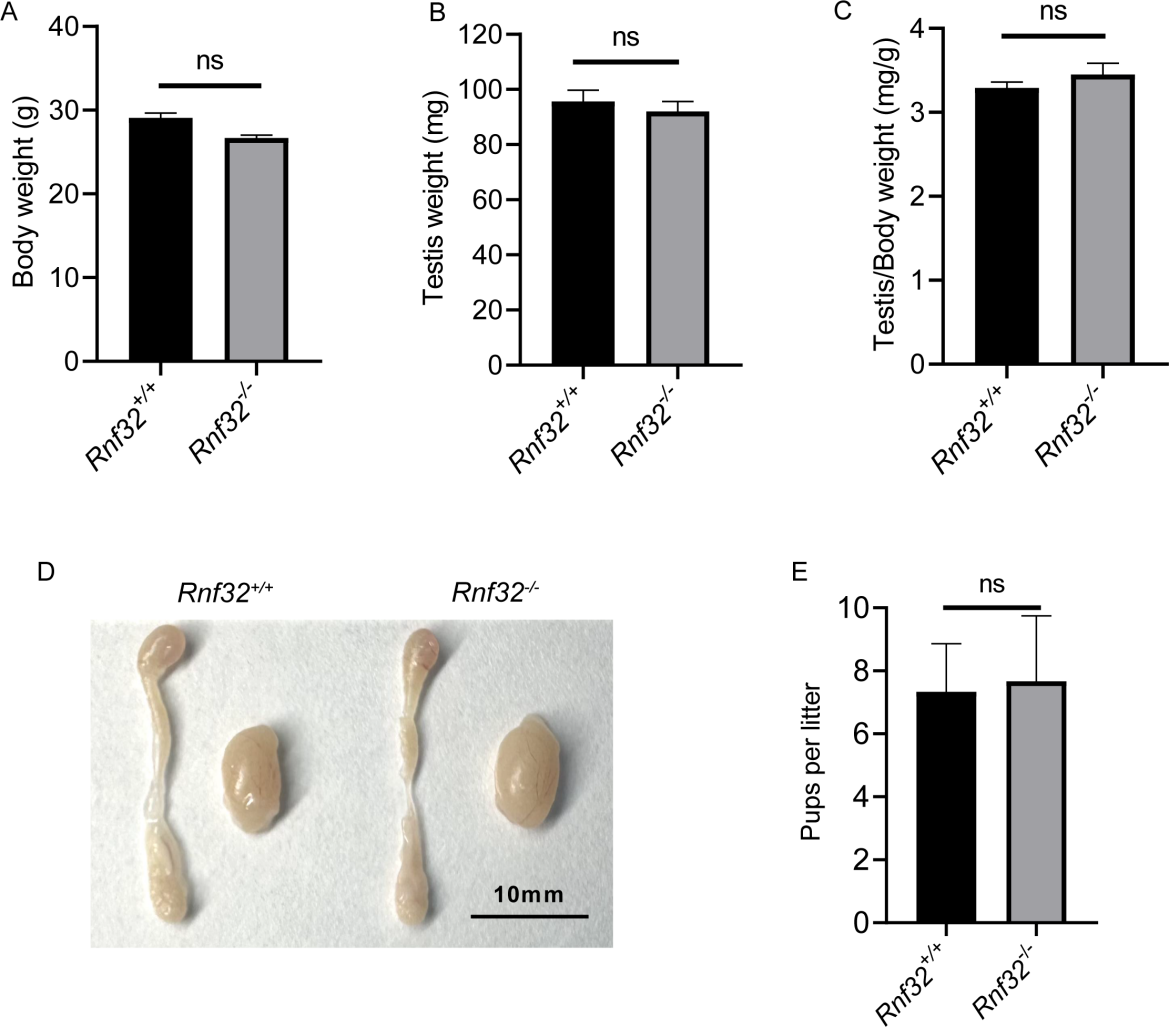
*Rnf32*^−/−^ mice are fertile. (A–C) Body weight, testis weight and testis/body weight ratio of adult *Rnf32*^+/+^ and *Rnf32*^−/−^ mice, *n* = 3, *P* > 0.05. (D) Representative images of testes and epididymides of adult (postnatal day 56) *Rnf32*^+/+^ and *Rnf32* ^−/−^ mice. (E) The litter size of *Rnf32*^+/+^ and * Rnf32*^−/−^ mice, *n* = 3, *P* > 0.05.

**Figure 3:**
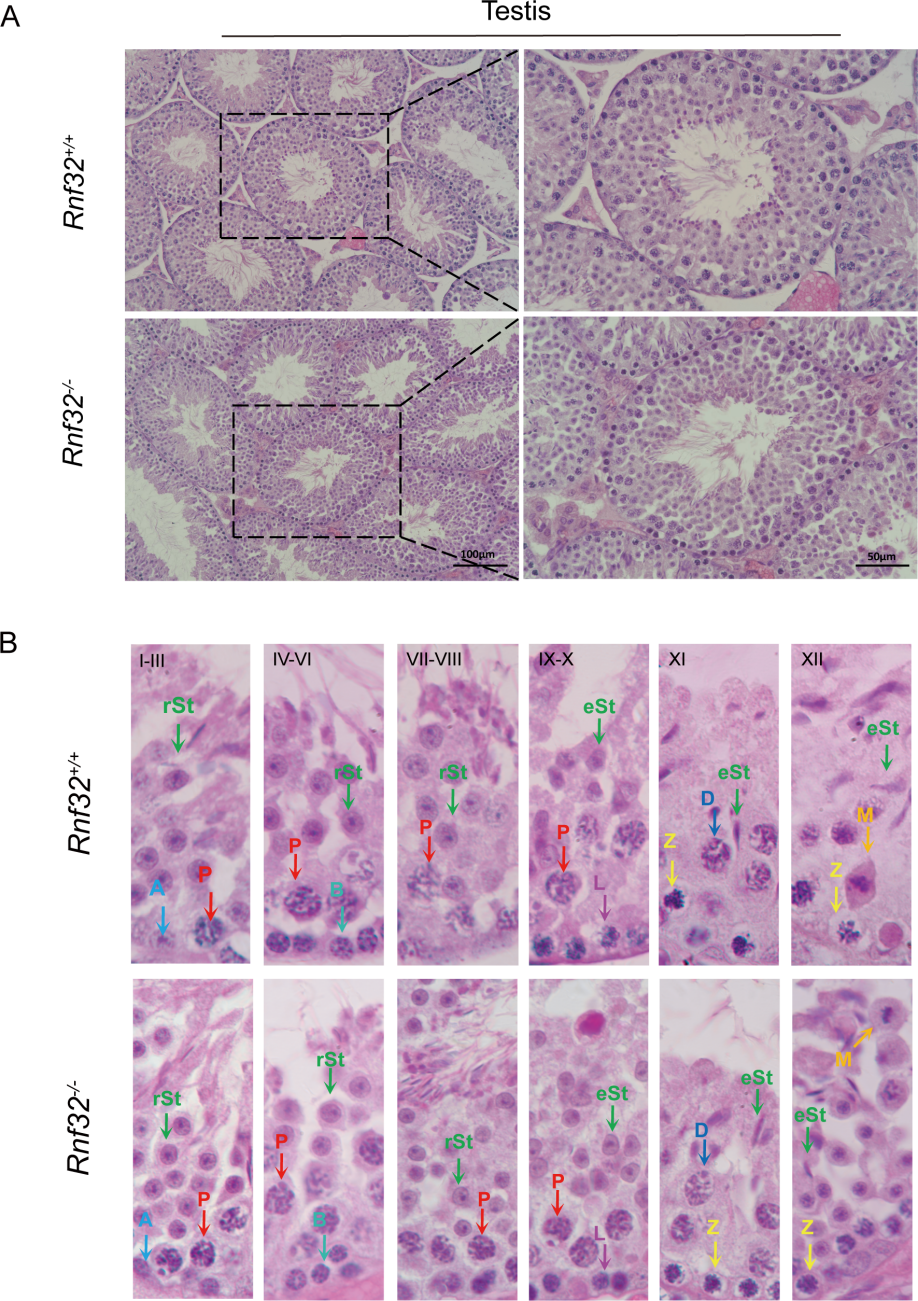
Spermatogenesis is normal in * Rnf32*^−/−^ mice. (A) H&E staining of the testis from adult *Rnf32*^+/+^ and *Rnf32*^−/−^ mice. (B) H&E staining of seminiferous tubules from adult *Rnf32*^+/+^ and *Rnf32*^−/−^ mice. L, leptotene spermatocyte; Z, zygotene spermatocyte; P, pachytene spermatocyte; M, meiotic spermatocyte; D, diplotene spermatocyte; rSt, round spermatid; eSt, elongated spermatid.

We conducted an examination of testicular morphology and testicular/body weight ratios in *Rnf32*-KO male mice, finding no statistically significant difference compared to the control group (Figs. 2E–2D). Following this, we carried out fertility tests to investigate the impact of *Rnf32* on mouse fertility, counting the pups and litters in both the experimental and control groups. The findings indicated that there was no statistically significant disparity in fertility between the WT and KO mice (Fig. 2E). In addition, we performed H&E staining on the testis, revealing normal spermatogenic cells of all stages of adult (postnatal 8w) *Rnf32*^−/−^ mice (Fig. 3A).

### The knockout of *Rnf32* does not impair spermatogenesis

**Figure 4:**
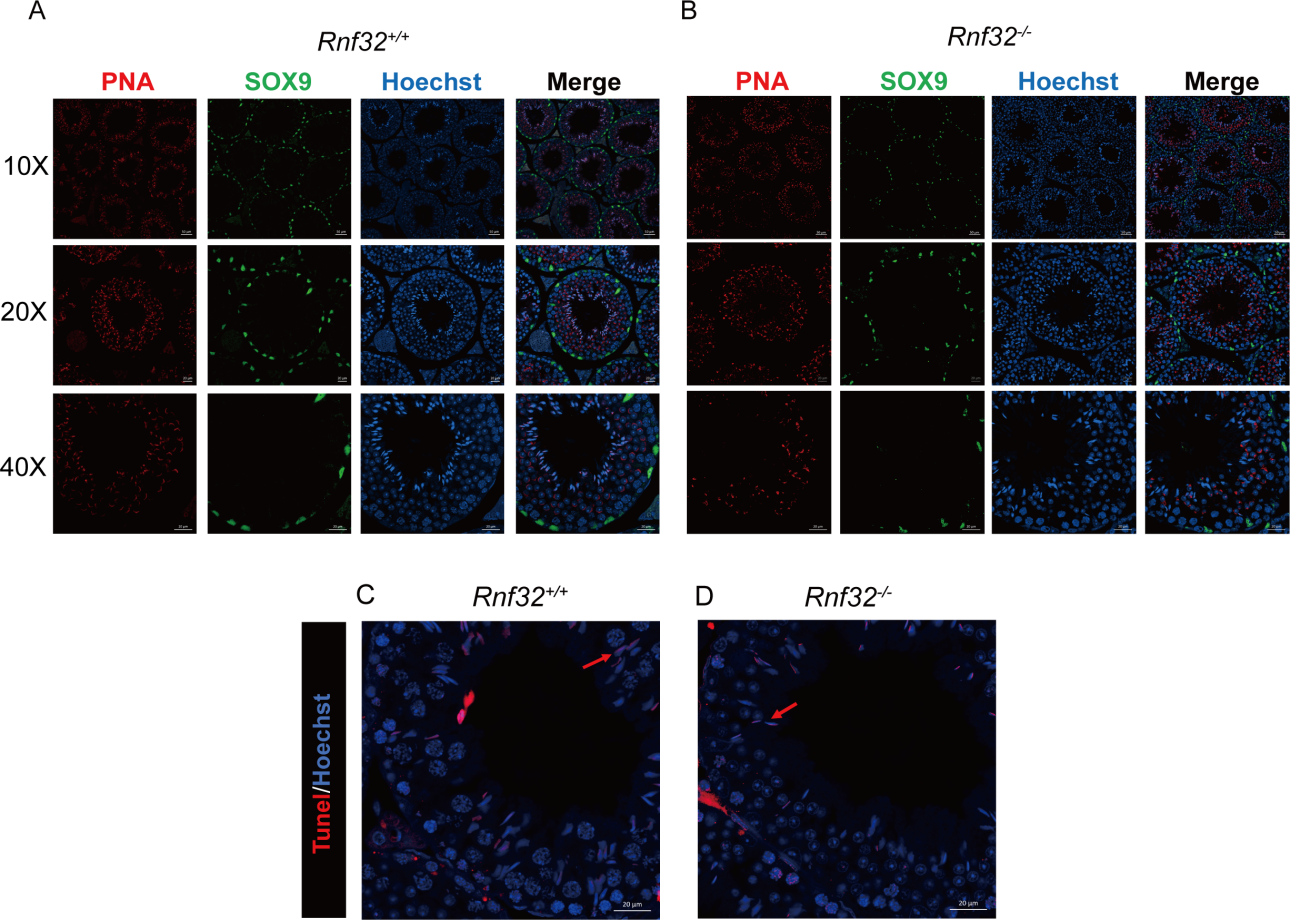
The knockout of *Rnf32* doesn’t affect spermatogenesis and apoptosis. Sertoli cells (SOX9) and spermatids (PNA) is comparable in adult (A) *Rnf32*^+/+^ and (B) *Rnf32*^−/−^ male mice testis. Apoptotic cells is comparable in adult (C) *Rnf32*^+/+^ and (D) *Rnf32*^−/−^ male mice testis.

Spermatogenesis, the fundamental biological process of germ cell differentiation, proceeds through precisely coordinated developmental stages in murine testes. The seminiferous epithelial cycle is classically categorized into 12 distinct phases based on spermatid maturation (Abe, Shen & Takano, 1991; Hess & Renato De Franca, 2008; Griswold, 2016), as defined by Bouin’s fixative (picric acid/formaldehyde/acetic acid)-preserved tissue analysis coupled with periodic acid-Schiff (PAS)-hematoxylin staining for precise staging of seminiferous epithelial cycles. To evaluate potential spermatogenic disruptions in *Rnf32*^+/+^ and *Rnf32*^−/−^ mice, we systematically analyzed germ cell cytoarchitecture across all 12 epithelial stages. Histological assessment revealed preserved seminiferous tubule organization in knockout testes, displaying complete germ cell lineages—including spermatogonia, spermatocytes, and elongating spermatids—with normal spatiotemporal progression of germ cell maturation indistinguishable from wild-type controls (Fig. 3B). Immunofluorescence quantification demonstrated comparable expression of SOX9, a Sertoli cell-specific transcription factor essential for spermatogonial niche maintenance, between adult *Rnf32*^+/+^ and *Rnf32*^−/−^ mice (Figs. 4A–4B). Quantitative data can be found in the Figs. S1A–S1B. Furthermore, TUNEL assay revealed statistically comparable rates of luminal germ cell apoptosis between adult *Rnf32* ^+/+^ and *Rnf32*^−/−^ mice (Figs. 4C–4D). Quantitative data can be found in the Figs. S1C–S1D.

### The knockout of *Rnf32* has no impact on sperm counts, morphology and motility

**Figure 5:**
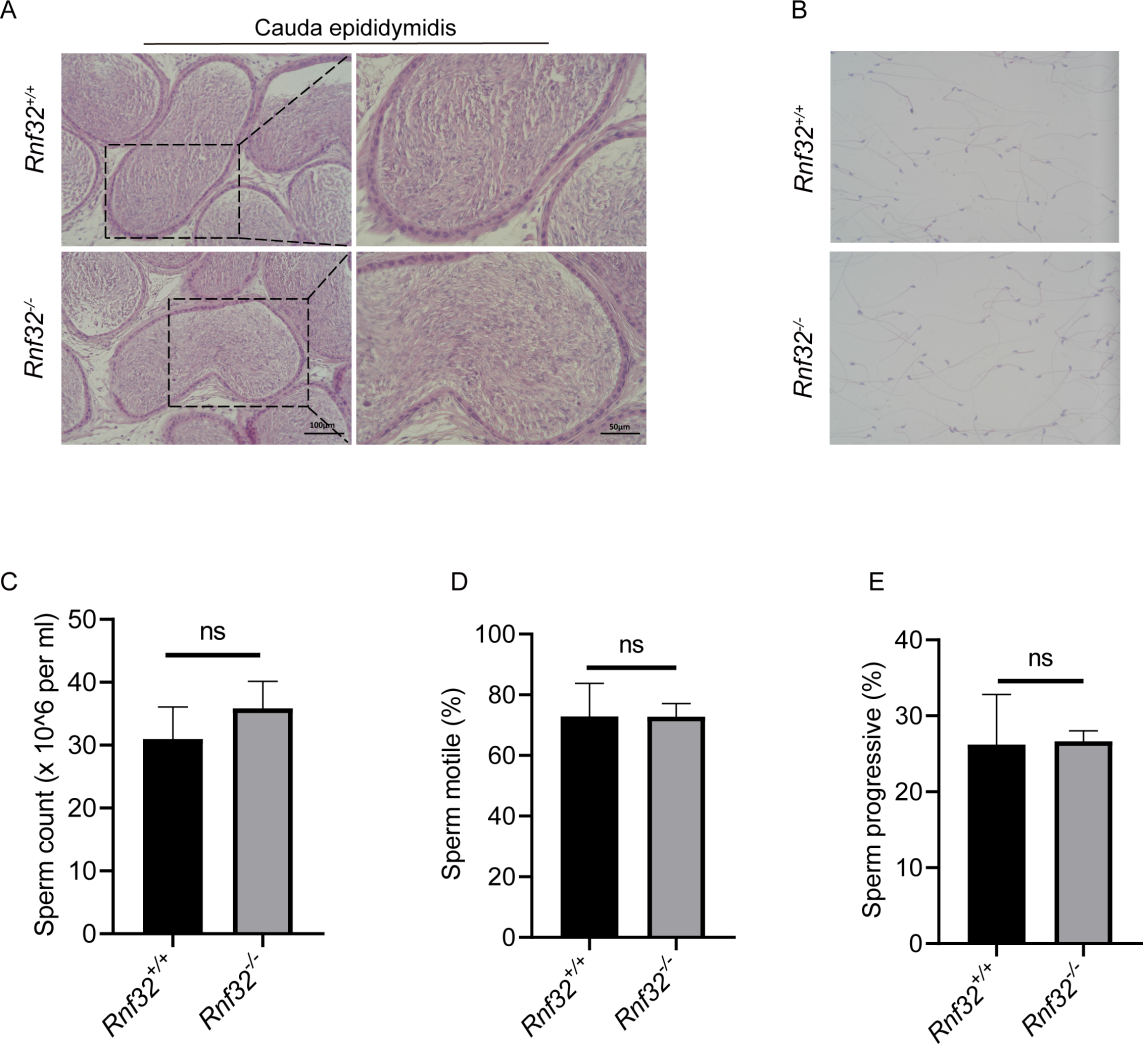
The knockout of *Rnf32* has no impact on sperm counts, morphology and motility. (A) H&E staining of cauda epididymidis from adult *Rnf32*^+/+^ and *Rnf32*^−/−^ mice. (B) H&E staining of sperm from adult *Rnf32*^+/+^ and *Rnf32*^−/−^ mice. (C–E) Sperm count, sperm motile and sperm progressive were assessed in adult *Rnf32*^+/+^ and *Rnf32*^−/−^ mice, *n* = 3, *P* > 0.05. All data are presented as means ± SEM, analyzed using a two-tailed unpaired *t*-test, ns indicates no difference.

To investigate the potential impact of *Rnf32* deficiency on sperm maturation in the epididymis, histopathological evaluation of the cauda epididymidis was performed. Comparative analysis demonstrated conserved sperm concentration in *Rnf32*^−/−^ epididymal luminal contents relative to wild-type controls (Fig. 5A). Ultrastructural assessment *via* differential interference contrast microscopy revealed preserved spermatozoal morphology in mutant mice, including typical head curvature and flagellar architecture (Fig. 5B). Quantitative data can be found in the (Fig. S1E). Computer-assisted semen analysis (CASA) was employed to quantify sperm kinematic parameters, such as count, motility, and progressive motility. The results indicated that there were no significant disparities in these metrics between *Rnf32* ^+/+^ and *Rnf32*^−/−^ mice (Figs. 5C–5E).

## Discussion

The utilization of genetically engineered mouse models (GEMMs) has profoundly enhanced our mechanistic understanding of reproductive developmental disorders associated with human infertility. Systematic interrogation of germline-specific gene ablation models has provided critical insights into the regulatory networks governing stem cell self-renewal, meiotic progression, and spermatogenic differentiation (Cooke & Saunders, 2002; Gilbert, Roof & Rajendra Kumar, 2018; Azhar et al., 2021). In this study, the generation of a CRISPR/Cas9-mediated *Rnf32* knockout murine model was achieved, enabling comprehensive phenotypic characterization. Comparative analysis revealed no significant genotypic differences in reproductive competence between *Rnf32*^−/−^ and wild-type (WT) males. These findings demonstrate that *Rnf32* is dispensable for murine male fertility, aligning with emerging evidence that numerous testis-enriched genes exhibit functional redundancy in reproductive contexts (Lv et al., 2022; Chen et al., 2022; Meng et al., 2023). Importantly, this study precludes RNF32 as a viable contraceptive target or monogenic infertility factor, thereby optimizing research resource allocation by eliminating nonproductive investigative pathways.

While *Rnf32* appears dispensable for baseline spermatogenesis, we hypothesize that Rnf32 may have a potential cytoprotective role in maintaining male fertility, particularly under conditions of environmental toxicity. Notably, certain genetic regulators, exemplified by the melanoma antigen (MAGE) family genes, demonstrate functional specialization in xenobiotic detoxification pathways. Although MAGE proteins are nonessential for physiological gametogenesis, they confer resilience against toxicant-induced spermatogenic impairment by modulating oxidative stress response cascades (Fon Tacer et al., 2019; Florke Gee et al., 2020; Li et al., 2023). Intriguingly, *Rnf32* shares evolutionary conserved structural motifs with these xenobiotic-responsive factors, suggesting possible involvement in analogous molecular mechanisms. We postulate that *Rnf32* may activate compensatory proteostatic networks under specific pathological conditions, thereby maintaining genomic integrity during spermatogonial stem cell proliferation and meiotic recombination. This hypothetical safeguard mechanism could explain the preserved fertility observed in *Rnf32*-deficient mice under controlled laboratory conditions while highlighting its potential clinical relevance in environmentally compromised reproductive scenarios. Another possibility is that functionally related genes may compensate for the loss of *Rnf32* upon its knockout. As Rnf32 is a member of the extensive RING finger protein family, characterized by conserved E3 ubiquitin ligase activity, it is plausible that structurally homologous family members exhibiting spatiotemporal expression overlap with Rnf32 may functionally compensate for the absence of Rnf32 through redundant or complementary enzymatic mechanisms. While our study employed a multi-modal approach to evaluate reproductive function—including fertility metrics (litter size) and spermatogenesis endpoints (sperm count, motility, histopathology)—the limited cohort size (*n* = 3 per group) constrains statistical power and generalizability. Additionally, our observations were confined to male mice.

## Conclusions

Despite robust *Rnf32* expression in murine testicular tissue, genetic ablation of this locus failed to induce discernible morphological abnormalities in testicular dimensions or spermatogenic cell populations. Crucially, comparative analysis of breeding performance revealed preserved reproductive capacity in *Rnf32*-KO males relative to wild-type counterparts.

## Supplemental Information

10.7717/peerj.19794/supp-1 Supplemental Information 1 Primers used in PCR,RT-PCR and RT-qPCR

10.7717/peerj.19794/supp-2 Supplemental Information 2 Raw CT values

10.7717/peerj.19794/supp-3 Supplemental Information 3 (A) PNA positive apoptotic cells counts in adult *Rnf32*^+/+^ and *Rnf32*^−/−^ mice, *n* = 3, *P* > 0.05 (B) SOX9 positive apoptotic cells counts in adult *Rnf32*^+/+^ and *Rnf32*^−/−^ mice, *n* = 3, *P* > 0.05. (C) TUNEL positive apoptotic tubule counts in adult *Rnf32*^+/+^ and *Rnf32*^−/−^ mice, *n* = 3, *P* > 0.05. (D) TUNEL positive apoptotic cells counts in adult *Rnf32*^+/+^ and *Rnf32*^−/−^ mice, *n* = 3, *P* > 0.05. (E) Percentage of abnormal sperm in adult * Rnf32* ^+/+^ and *Rnf32*^−/−^ mice; *n* = 3, *P* > 0.05.

10.7717/peerj.19794/supp-4 Supplemental Information 4 Original Figs. 3D–3E

10.7717/peerj.19794/supp-5 Supplemental Information 5 Raw data

10.7717/peerj.19794/supp-6 Supplemental Information 6 Raw data analysis Open with GraphPad Prism (https://www.graphpad-prism.cn).

10.7717/peerj.19794/supp-7 Supplemental Information 7 Author checklist

10.7717/peerj.19794/supp-8 Supplemental Information 8 MIQE checklist

## Additional Information and Declarations

### Data Availability

The following information was supplied regarding data availability: The raw measurements are available in the Supplementary File.

## References

[ref-1] Abe K, Shen LS, Takano H (1991). The cycle of the seminiferous epithelium and stages in spermatogenesis in dd-mice. [Hokkaido Igaku Zasshi] The Hokkaido Journal of Medical Science.

[ref-2] Azhar M, Altaf S, Uddin I, Cheng J, Wu L, Tong X, Qin W, Bao J (2021). Towards post-meiotic sperm production: genetic insight into human infertility from mouse models. International Journal of Biological Sciences.

[ref-3] Barratt CLR, Björndahl L, De Jonge CJ, Lamb DJ, Osorio Martini F, McLachlan R, Oates RD, Van der Poel S, St John B, Sigman M, Sokol R, Tournaye H (2017). The diagnosis of male infertility: an analysis of the evidence to support the development of global WHO guidance-challenges and future research opportunities. Human Reproduction Update.

[ref-4] Chasapis CT, Spyroulias GA (2009). RING finger E(3) ubiquitin ligases: structure and drug discovery. Current Pharmaceutical Design.

[ref-5] Chen A, Ji C, Li C, Brand-Saberi B, Zhang S (2024). Multiple transcriptome analyses reveal mouse testis developmental dynamics. BMC Genomics.

[ref-6] Chen Z, Ma D, Jin T, Yu Z, Li J, Sun Q, Li Z, Du Z, Liu R, Li Y, Luo M (2022). Fbxw17 is dispensable for viability and fertility in mice. Molecular Biology Reports.

[ref-7] Chen Y, Zheng Y, Gao Y, Lin Z, Yang S, Wang T, Wang Q, Xie N, Hua R, Liu M, Sha J, Griswold MD, Li J, Tang F, Tong M-H (2018). Single-cell RNA-seq uncovers dynamic processes and critical regulators in mouse spermatogenesis. Cell Research.

[ref-8] Cooke HJ, Saunders PTK (2002). Mouse models of male infertility. Nature Reviews. Genetics.

[ref-9] Cooper TG (2007). Sperm maturation in the epididymis: a new look at an old problem. Asian Journal of Andrology.

[ref-10] Dacheux J-L, Dacheux F (2014). New insights into epididymal function in relation to sperm maturation. Reproduction.

[ref-11] De Kretser DM, Baker HW (1999). Infertility in men: recent advances and continuing controversies. The Journal of Clinical Endocrinology and Metabolism.

[ref-12] Florke Gee RR, Chen H, Lee AK, Daly CA, Wilander BA, Fon Tacer K, Potts PR (2020). Emerging roles of the MAGE protein family in stress response pathways. The Journal of Biological Chemistry.

[ref-13] Fon Tacer K, Montoya MC, Oatley MJ, Lord T, Oatley JM, Klein J, Ravichandran R, Tillman H, Kim M, Connelly JP, Pruett-Miller SM, Bookout AL, Binshtock E, Kamiński MM, Potts PR (2019). MAGE cancer-testis antigens protect the mammalian germline under environmental stress. Science Advances.

[ref-14] Gilbert SB, Roof AK, Rajendra Kumar T (2018). Mouse models for the analysis of gonadotropin secretion and action. Best Practice & Research. Clinical Endocrinology & Metabolism.

[ref-15] Gong W, Pan L, Lin Q, Zhou Y, Xin C, Yu X, Cui P, Hu S, Yu J (2013). Transcriptome profiling of the developing postnatal mouse testis using next-generation sequencing. Science China. Life Sciences.

[ref-16] Griswold MD (2016). Spermatogenesis: the commitment to meiosis. Physiological Reviews.

[ref-17] Guo R, Yu Z, Guan J, Ge Y, Ma J, Li S, Wang S, Xue S, Han D (2004). Stage-specific and tissue-specific expression characteristics of differentially expressed genes during mouse spermatogenesis. Molecular Reproduction and Development.

[ref-18] Hackstein JH, Hochstenbach R, Pearson PL (2000). Towards an understanding of the genetics of human male infertility: lessons from flies. Trends in Genetics: TIG.

[ref-19] Hess RA, Renato De Franca L (2008). Spermatogenesis and cycle of the seminiferous epithelium. Advances in Experimental Medicine and Biology.

[ref-20] Ickowicz D, Finkelstein M, Breitbart H (2012). Mechanism of sperm capacitation and the acrosome reaction: role of protein kinases. Asian Journal of Andrology.

[ref-21] Li H, Huo Y, He X, Yao L, Zhang H, Cui Y, Xiao H, Xie W, Zhang D, Wang Y, Zhang S, Tu H, Cheng Y, Guo Y, Cao X, Zhu Y, Jiang T, Guo X, Qin Y, Sha J (2022). A male germ-cell-specific ribosome controls male fertility. Nature.

[ref-22] Li N, Yu J, Zhou M, Qiu F, Wang X, Wang Z (2023). MAGE-B4, a binding partner of PRAMEF12, is dispensable for spermatogenesis and male fertility in mice. Biochemical and Biophysical Research Communications.

[ref-23] Lv C, Xiong M, Guo S, Gui Y, Liu X, Wang X, Wu Y, Feng S, Zhang J, Zhang Y, Liu Y, Qin W, Yuan S (2022). WDFY1, a WD40 repeat protein, is not essential for spermatogenesis and male fertility in mice. Biochemical and Biophysical Research Communications.

[ref-24] McLachlan RI, Rajpert-De Meyts E, Hoei-Hansen CE, De Kretser DM, Skakkebaek NE (2007). Histological evaluation of the human testis–approaches to optimizing the clinical value of the assessment: mini review. Human Reproduction.

[ref-25] Meng Z, Qiao Y, Xue J, Wu T, Gao W, Huang X, Lv J, Liu M, Shen C (2023). Slc26a1 is not essential for spermatogenesis and male fertility in mice. PeerJ.

[ref-26] Nishimura H, L’Hernault SW (2017). Spermatogenesis. Current Biology: CB.

[ref-27] Saint-Dizier M, Mahé C, Reynaud K, Tsikis G, Mermillod P, Druart X (2020). Sperm interactions with the female reproductive tract: a key for successful fertilization in mammals. Molecular and Cellular Endocrinology.

[ref-28] Van Baren MJ, Van der Linde HC, Breedveld GJ, Baarends WM, Rizzu P, de Graaff E, Oostra BA, Heutink P (2002). A double RING-H2 domain in RNF32, a gene expressed during sperm formation. Biochemical and Biophysical Research Communications.

[ref-29] Yu Z, Guo R, Ge Y, Ma J, Guan J, Li S, Sun X, Xue S, Han D (2003). Gene expression profiles in different stages of mouse spermatogenic cells during spermatogenesis. Biology of Reproduction.

[ref-30] Zou Q, Yang L, Shi R, Qi Y, Zhang X, Qi H (2021). Proteostasis regulated by testis-specific ribosomal protein RPL39L maintains mouse spermatogenesis. iScience.

