# Supplementary material for: Rnf32 is not essential for spermatogenesis and male fertility in mice
Source: PeerJ. 2025 Jul 30;13:e19794. doi: 10.7717/peerj.19794 (PMC12317687; doi:10.7717/peerj.19794)
Supplement: Supplemental Information 1 [file peerj-13-19794-s001.pdf]

Table S1. Primers used in PCR,RT-PCR and RT-qPCR

Primer name	Primer name(5'-3')	Expected product size (bp)
Rnf32-RT-Qpcr-F	GTTGCCTTACAGGACCACCTA	
Rnf32-RT-Qpcr-R	CTATGATGGCTGTTGCATCTCTT	133
Rnf32-pcr-F	GCACGGCAGTCTTATACCTAGCAT	
Rnf32-pcr-R	GTCCTCTTACCCACCAAACCATCC	539
18S-F	TAACGAACGAGACTCTGGCAT	
18S-R	CGGACATCTAAGGGCATCACAG	138
