# Supplementary figures and images for: Rnf32 is not essential for spermatogenesis and male fertility in mice
Source: PeerJ. 2025 Jul 30;13:e19794. doi: 10.7717/peerj.19794 (PMC12317687; doi:10.7717/peerj.19794)

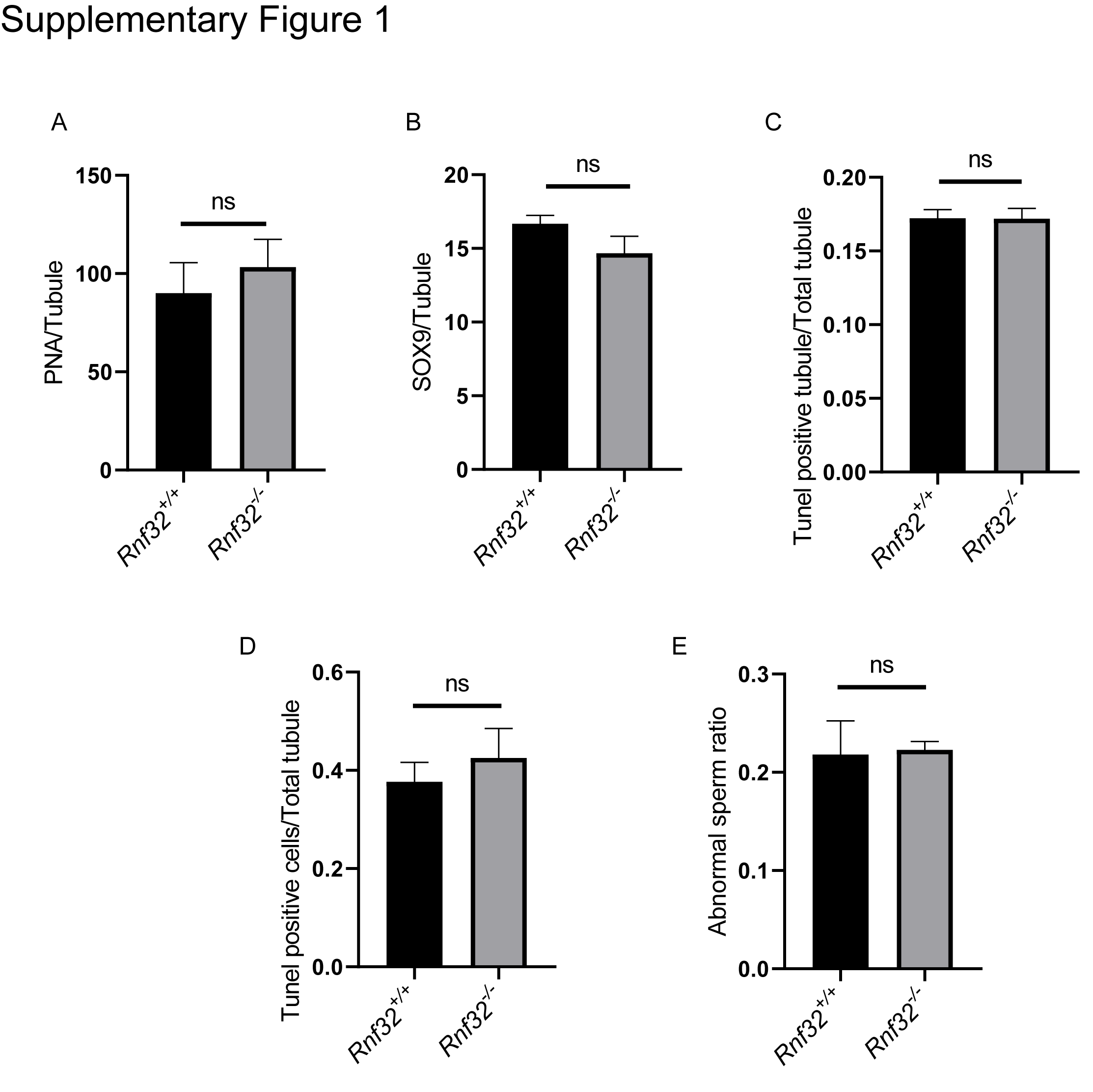

Supplement: Supplemental Information 3 — (B) SOX9 positive apoptotic cells counts in adult Rnf32+/+ and Rnf32−/− mice, n = 3, P > 0.05. (C) TUNEL positive apoptotic tubule counts in adult Rnf32+/+ and Rnf32−/− mice, n = 3, P > 0.05. (D) TUNEL positive apoptotic cells counts in adult Rnf32+/+ and Rnf32−/− mice, n = 3, P > 0.05. (E) Percentage of abnormal sperm in adult Rnf32 +/+ and Rnf32−/− mice; n = 3, P > 0.05. [file peerj-13-19794-s003.png]
